# Deep learning for the PSIPRED Protein Analysis Workbench

**DOI:** 10.1093/nar/gkae328

**Published:** 2024-05-15

**Authors:** Daniel W A Buchan, Lewis Moffat, Andy Lau, Shaun M Kandathil, David T Jones

**Affiliations:** UCL Bioinformatics Group, Department of Computer Science, University College London, London, WC1E 6BT, UK; UCL Bioinformatics Group, Department of Computer Science, University College London, London, WC1E 6BT, UK; UCL Bioinformatics Group, Department of Computer Science, University College London, London, WC1E 6BT, UK; UCL Bioinformatics Group, Department of Computer Science, University College London, London, WC1E 6BT, UK; UCL Bioinformatics Group, Department of Computer Science, University College London, London, WC1E 6BT, UK

## Abstract

The PSIRED Workbench is a long established and popular bioinformatics web service offering a wide range of machine learning based analyses for characterizing protein structure and function. In this paper we provide an update of the recent additions and developments to the webserver, with a focus on new Deep Learning based methods. We briefly discuss some trends in server usage since the publication of AlphaFold2 and we give an overview of some upcoming developments for the service. The PSIPRED Workbench is available at http://bioinf.cs.ucl.ac.uk/psipred.

## Graphical Abstract

**Graphical Abstract:**
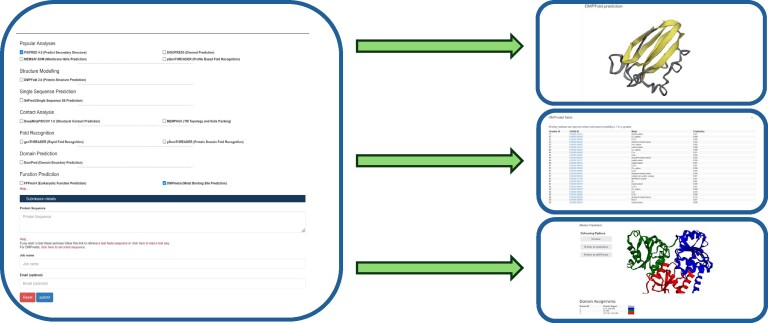

## Introduction

The PSIPRED Workbench is part of a worldwide ecosystem of Bioscience data repositories and web services. These cover primary data repositories such as the NCBI, EBI and RCSB PDB (1–3), derived data resources such as STRING, CATH, KEGG, InterPro and UniProt (4–8), and webservices such as EBI Webservices, NCBI Webservices, among a great many others. A large number of tools and services available as code and webservices can be discovered via the Elixir BioTools web site (https://bio.tools/) (9).

We have been developing the PSIPRED Workbench for nearly 25 years. Our webservices offer a variety of machine learning-based tools focussed on characterising structural and functional features of proteins. In recent years, we have made significant headway in integrating new deep-learning based tools and techniques. In 2018, we replaced every line of code in our webserver and significantly improved both tool run times and presentation. Since then, we have seen peak annual usage rise to the order of 350 000 analyses per year.

Our services critically rely on underlying datasets from UniRef (10) and the PDB. Like all bioscience data resources, we have witnessed exponential growth in the size of these published datasets, which creates many computational challenges for bioinformatics tools and web servers. Many of our methods function by analysing evolutionary information in protein families, and protein database searching forms a critical first step in most of our tools. As the size of these resources grows, the runtimes for such analysis lengthens. To tackle this, we are increasingly looking to deep learning. Through careful model training, it is possible to embed protein sequence information such as evolutionary relationships between residues within the weights of a neural network (11,12). Consequently, we can use these and similar embeddings alongside novel deep learning-based methods and forgo the need for computationally expensive protein database searches while still producing accurate predictions of protein features that rely on evolutionary information.

### New methods

Since 2019, we have published a number of new methods in the UCL Bioinformatics Group and have made some of these available online via the PSIPRED Workbench. Below we give a summary of the methods we have added to the webserver.

### S4PRED

S4PRED (13) is a state-of-the-art single-sequence protein secondary structure prediction method. It is used to provide accurate secondary structure modelling for a challenging but important class of proteins, namely single orphan proteins, which have no detectable sequence relatives in current databases. Accordingly, the model takes only a protein's amino acid sequence as input, with no additional homology information, and subsequently returns 3-state secondary structure predictions for the sequence. Similarly to PSIPRED, S4PRED prediction results comprise a confidence score, a cartoon representation, 3-state prediction assignment, and the original amino acid sequence.

**Figure 1.**
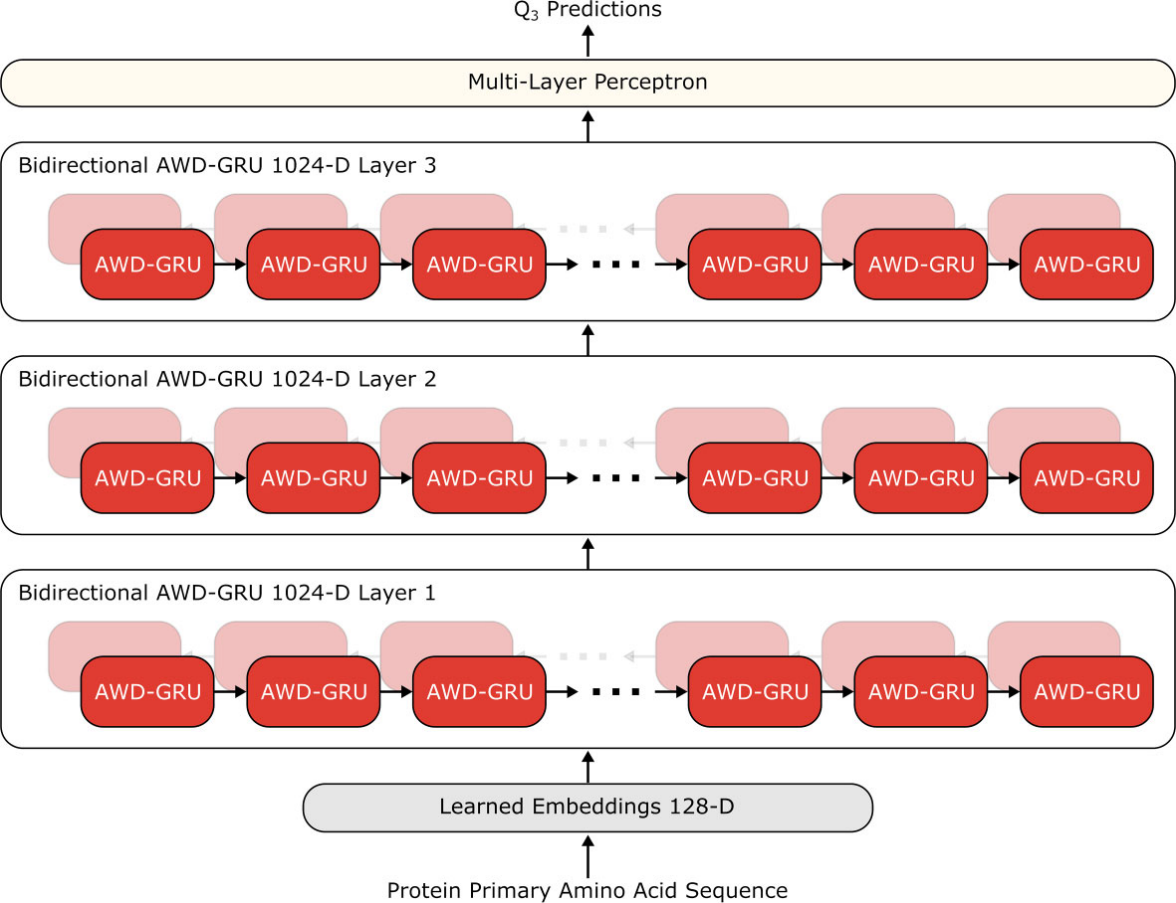
The neural network architecture of the S4PRED model. Single protein sequences are given as input to the model. Each amino acid in a given sequence is first dynamically replaced with a 128-dimensional vector embedding that is learned during training. The sequence of embeddings is then fed to a 1024-dimensional bidirectional recurrent neural network (RNN), termed the first layer. The specific RNN architecture used is the Averaged stochastic gradient descent Weight-Dropped Gated Recurrent Unit (AWD-GRU) (38,39). The RNN output is fed through two more layers and then a final multilayer perceptron which transforms the output to 3-dimensional probability predictions for each Q3 class.

The model's architecture is an ensemble of five 3-layered recurrent deep neural networks (see Figure 1). It is trained using a semi-supervised learning approach to massively supplement the available number of protein sequences that can be trained on. This results in a training set in excess of a million examples. This set combines real-labelled examples, where a sequence and its secondary structure are known, and artificially labelled examples, where only the primary amino acid sequence is known. S4PRED has a Q3 secondary structure prediction accuracy of 75.3%. This is a significant improvement over our cutting edge PSIPRED method, which achieves a Q3 accuracy of 70.6% when tested on single sequences without any provided homology information. For secondary prediction tasks typical run times are of the order of seconds on contemporary CPUs.

### Merizo

Merizo is a deep learning-based method for protein domain segmentation (14). The method operates directly on structures and can produce accurate domain assignments even for discontinuous domains, as well as for predicted models from AlphaFold2 (15) which may feature long stretches of unstructured, non-domain residues.

The network of Merizo is based on an encoder-decoder architecture that utilises the invariant point attention module (introduced in AlphaFold2) to encode a structure and its sequence into an embedding. This embedding is then decoded using a Masked Transformer Decoder (16) to assign individual residues into domains in a bottom-up manner. Merizo is trained using an affinity learning strategy (17), wherein the network learns to cluster together embeddings of residues that belong to the same domain.

In a benchmark study on PDB structures, Merizo outperforms several state-of-the-art domain assignment methods, including both deep learning and non-deep learning-based methods, producing accurate assignments that are well-aligned with those documented in the CATH database. As a proof of concept, Merizo has also been applied to the human proteome, identifying over 40 000 domains that can be matched to known folds in CATH, while requiring only a fraction of the time needed by other methods.

**Figure 2.**
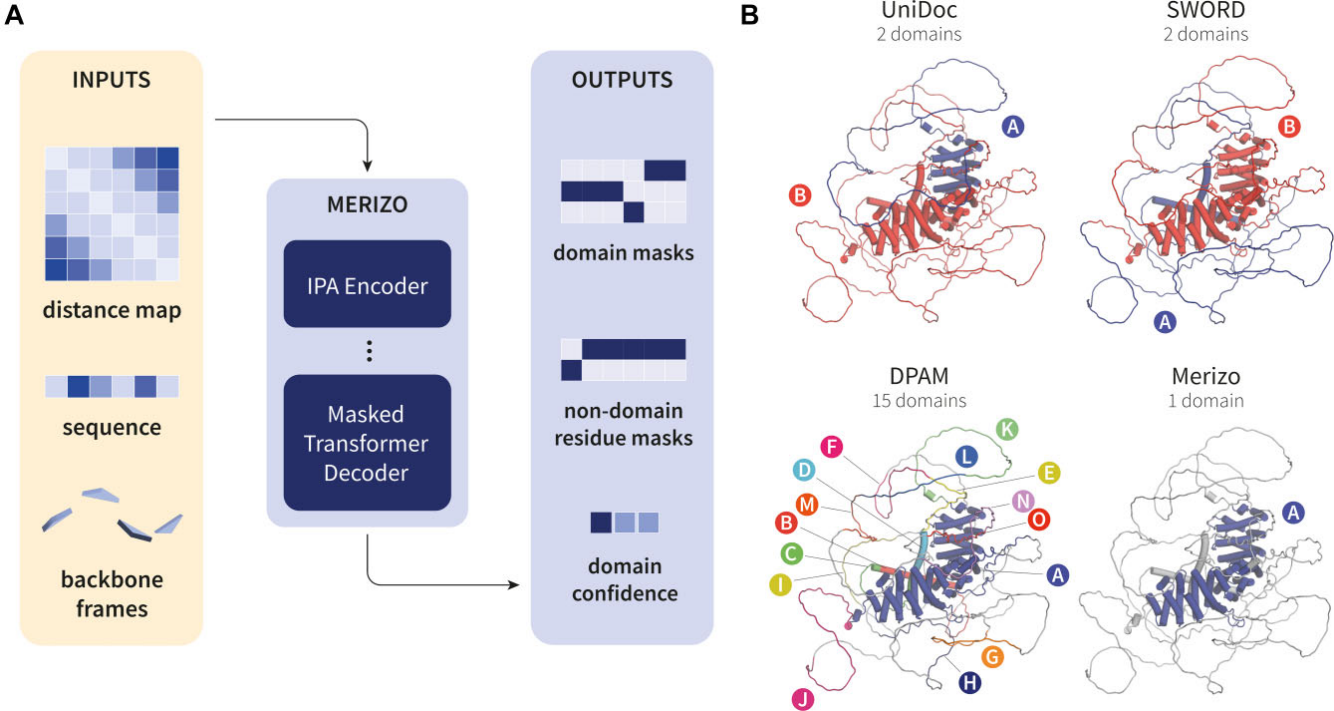
Overview of Merizo. (**A**) Summary of inputs and outputs. Merizo takes the C-alpha distance map along with the amino acid sequence and backbone frames (calculated as per Jumper *et al.*, 2021) as input. Inputs are fed into the IPA encoder which generates an embedding of the structure. The embedding is decoded by a masked transformer decoder to generate domain and non-domain residue masks, along with a confidence estimate for each predicted domain. (**B**) Example of domain assignments by several methods including Merizo on AlphaFold2 model AF-Q9UQB3-F1-model_v4. Methods include UniDoc (40), SWORD (41) and DPAM (42). Assigned domains are individually coloured and labelled from A-O. (Figure adapted from Lau *et al*, 14).

Typical prediction times are around 1 second on modern CPUs and nearly an order of magnitude faster when using a GPU. Predictions are also shown to be highly accurate; Merizo achieves a median MCC score of approximately 1.0 when benchmarking predictions against known CATH or ECOD domain boundaries in multidomain proteins, and has a mean absolute error of ∼0.3 when predicting the number of domains within a chain correctly, outperforming other leading methods at this task. A brief overview of Merizo's inputs, outputs and illustrative performance to similar methods is given in Figure 2.

### DMPFold2

**Figure 3.**
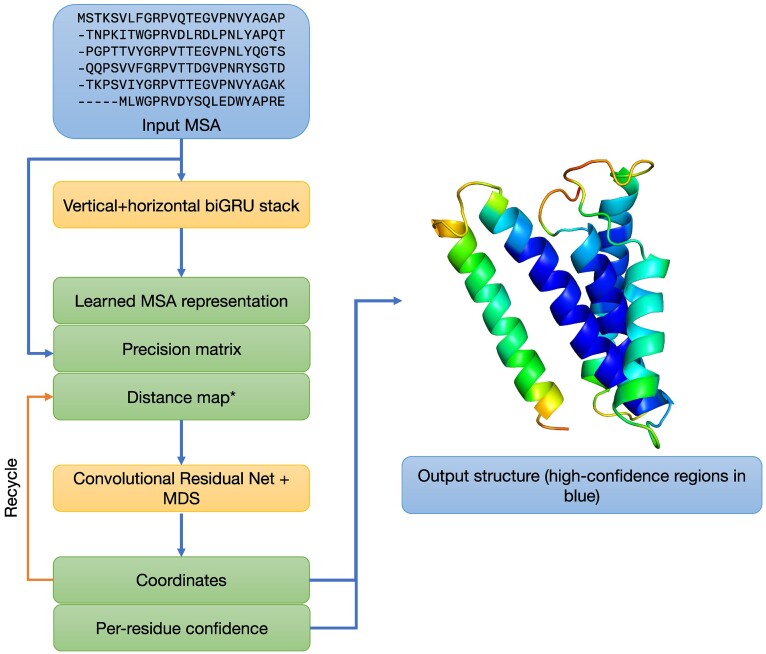
Overview of protein tertiary structure prediction using DMPfold2. *Coordinates produced at the end of the network can optionally be converted into a distance map and used to refine predictions in an iterative fashion. This distance map is zeroed out in the first iteration.

DMPfold2 (18) predicts the tertiary structure of single protein chains starting from amino acid sequence. It improves upon its predecessor DMPfold (19) in terms of both accuracy and speed of execution. The high speed of execution is enabled by a novel neural network architecture that takes as input a multiple sequence alignment (MSA) of the target protein sequence, and outputs the coordinates of C-alpha atoms of the main chain as direct outputs of the neural network. Alongside the coordinates, the network also predicts a per-residue confidence score. To predict the structure, the amino acids in the input MSA is first encoded as integers and then processed by a sequence of bidirectional Gated Recurrent Unit (biGRU) networks, the first operating on columns of the MSA to produce per-column representations. The second biGRU takes these representations as input and processes them in the horizontal direction to produce a final representation. This representation is combined along with a fast approximation of the residue precision matrix and is fed to a stack of residual convolutional layers. The output from this stack is then treated as a distance matrix and subjected to a differentiable multidimensional scaling procedure to recover the coordinates of the C-alpha atoms. The remaining main-chain atoms are added using the catomain procedure (20) and sidechain atoms can subsequently be added using tools such as SCWRL (21). Once a set of C-alpha coordinates have been generated, they can be converted into a pairwise distance map and used as an additional input to the network, and thus predictions can be recycled for iterative refinement. An overview of the method is presented in Figure 3.

Although not as accurate as AlphaFold2 and RoseTTAFold (22), DMPfold2 is orders of magnitude faster than these methods, and has considerably lower resource requirements. The former two methods require the use of GPU AI accelerators to achieve reasonable runtimes, however DMPfold2 is fast enough to be run on CPUs with runtimes ranging from seconds to a few minutes once the input MSA has been generated. On GPUs, DMPfold2 was shown to be roughly 2 orders of magnitude faster than AlphaFold2 in a head-to-head comparison.

### DMPmetal

DMPmetal is a deep learning-based method for predicting metal binding sites from amino acid sequences. It follows the approach of using a large (1.2 billion parameter) pre-trained transformer encoder protein language model (pLM) to embed the target sequences and to provide the features for simple feed-forward classifier. One difference from many other pLMs is that the DMPmetal pLM was jointly pre-trained on both sequence and structures through training on the UniRef50 subset of the AlphaFold Database (23). From a user perspective, the input to the model is a protein sequence, and the output probabilities relate to each of the 29 CHEBI metal codes. This model was ranked 1^st^ in the UniProt Metal Binding Site Machine Learning Challenge held in 2022, and was trained on the organizers’ provided NEG_TRAIN and POS_TRAIN_FULL datasets, based on curated UniProt annotations (http://insideuniprot.blogspot.com/2022/02/the-uniprot-metal-binding-site-machine.html).

### Available methods

The PSIPRED Workbench offers a number of analysis methods. We summarize these and their principal publication in Table 1.

**Table 1.** Methods available via the PSIPRED workbench

Method	Summary	Citation
PSIPRED 4.0	Secondary structure prediction	Protein secondary structure prediction based on position-specific scoring matrices (24)
DISOPRED3	Disordered residue prediction	DISOPRED3: precise disordered region predictions with annotated protein-binding activity (25)
MEMSAT-SVM	Membrane helix prediction	Predicting transmembrane helix packing arrangements using residue contacts and a force-directed algorithm (26)
GenTHREADER, pGenTHREADER & pDomTHREADER	Fold recognition	pGenTHREADER and pDomTHREADER: new methods for improved protein fold recognition and superfamily discrimination (27)
DeepMetaPSICOV 1.0	Structural contact prediction	Prediction of interresidue contacts with DeepMetaPSICOV in CASP13 (28)
DomPred	Protein domain boundary prediction	Computer-assisted protein domain boundary prediction using the DomPred server (29)
DMPFold 2.0	Fast and Accurate Deep Learning Based protein structure prediciton	Ultrafast end-to-end protein structure prediction enables high-throughput exploration of uncharacterized proteins (18)
FFPred3	GO Term functional prediction	FFPred 3: feature-based function prediction for all Gene Ontology domains (30)
Metsite	Metal binding site prediction	Predicting metal-binding site residues in low-resolution structural mode (31)
HSPred	Protein-protein interaction hotspot prediction	Predictions of hot spot residues at protein-protein interfaces using support vector machines (32)
MEMEMBED	Membrane protein orientation prediction	Membrane protein orientation and refinement using a knowledge-based statistical potential (33)
Merizo	Deep Learning base structural domain segmentation	Merizo: a rapid and accurate protein domain segmentation method using invariant point attention (14)
S4PRED	Single Sequence Protein secondary Structure Prediction	Increasing the accuracy of single sequence prediction methods using a deep semi-supervised learning framework (13)
DMPmetal	Metal binding site prediction for protein sequences	Manuscript in preparation

## Retired methods

As science progresses, some of our older methods become obsolete. We now take the approach that prediction tools on our webserver which consistently see fewer than 1000 requests per year become candidates to be retired. We then assess these methods to establish if they have become obsolete; that is, they have either been replaced by a method within our group or have been made obsolete by other advances or tools that have emerged within protein bioinformatics. This year, we have chosen to retire our methods BioSerf and DomSerf, which were fully automated homology modelling packages which generated protein structure predictions. We see that these are amply superseded by the performance of methods such as DMPFold2 and AlphaFold2. Code for these methods will remain available in our public code repository.

### Trends In usage since 2018

**Figure 4.**
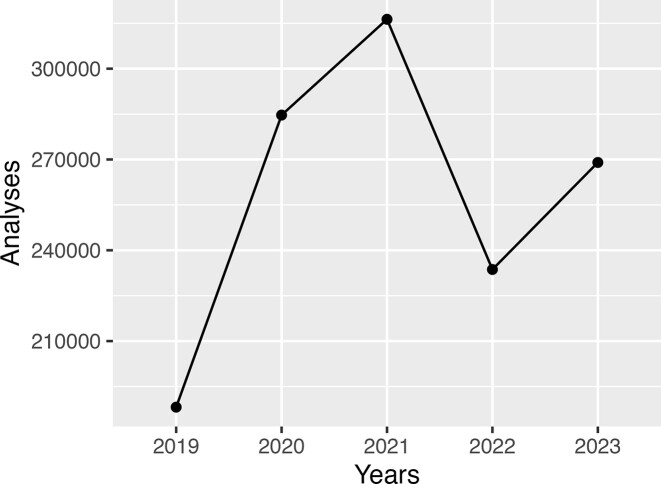
Total number of predictive analysis tasks run by the PSIPRED Workbench in the years 2019 to 2023. Y-axis is truncated.

**Figure 5.**
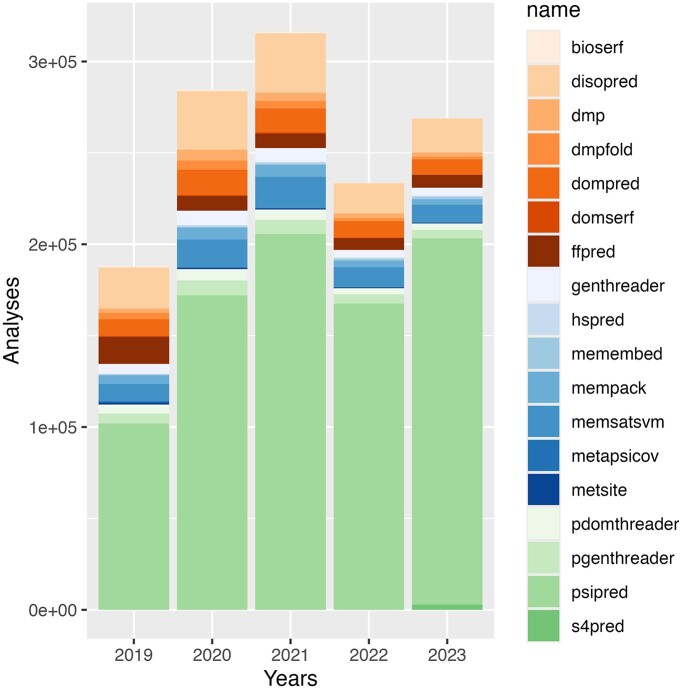
Bar chart of the proportion of predictive analyses jobs requested by user, coloured by predictive method. Data is broken down by year.

Figure 4 shows the trends in usage of the PSIPRED web server since 2018, when our faster and more user-friendly web site was first launched. In the two years immediately following this, we saw substantial growth in the number of jobs submitted, aided no doubt by the increasing interest and use of novel bioinformatics tools in general during this time. However, in 2022, we saw a sharp decline in job counts, which we attribute to the availability of AlphaFold2 (15) and the associated AlphaFold structure database (23), which, at least in theory, would obviate the need for secondary structure and other predictions. Nevertheless, in 2023, submission counts for secondary structure prediction rebounded to their pre-2022 levels (see Figure 5). We suspect that over time, researchers became more familiar with the limitations of pre-calculated structure models (as observed by others, 34,35), and the additional difficulty in handling 3D structural data when only a protein sequence annotation is required. There clearly remains a demand for methods that can either corroborate the predictions made by structure modelling methods, or that can provide data that can be interpreted rapidly and more directly, for example in evaluating point mutants of some protein sequences.

### Site reliability and server developments

The principal focus of our web site development work since 2018 has been a new JavaScript front end code base. Our previous web site was implemented using the Ractive JavaScript framework (https://ractive.js.org/). This was an excellent choice in 2016 for rapidly prototyping our new web site, but as time passed and the site grew in complexity, the code became quite labyrinthine and hard to maintain. Since then, we have ported the entire website to React (https://react.dev/). We believe this gives a number of benefits; React is highly opinionated about application structure, and this should reduce any tendency towards writing unmaintainable, spaghetti code. React has also emerged as something of an industry standard for designing dynamic web applications so we anticipate that the framework will be well-supported for years to come.

Alongside this work on the web code we have recently upgraded the web server hardware. The old web server hardware had reached end-of-life and has been replaced with three new modern server machines. Alongside this, we will be adding a further data processing machine with 48 cores. This should provide sufficient additional capacity for the web site's developments in the years to come.

In our prior 2018/2019 web server publication (36), we replaced the entire code base for the website and installed new data analysis pipeline middleware. Since then, we are pleased to report that the webserver has experienced no downtime due to software failures. All server downtime has been due to scheduled hardware maintenance or unplanned hardware failures, such as power outages. Both our webserver and middleware code display excellent reliability with little need for ongoing maintenance. However, we do see a number of analyses fail. Sometimes our predictive methods have bugs or perhaps cannot handle certain edge cases; and on occasion users are able to submit erroneous input data. Nevertheless, the number of such failed jobs is small, at only around 3000 failures per year, typically <1% of all analyses each year.

## Discussion

We’ve reviewed in this paper some recent updates to our web services. Looking to the future, with the advent of AlphaFold2 and accurate structural modelling, we anticipate that a structural approach to protein bioinformatics will become increasingly common. With this in mind, our future developments for the service will focus on providing a novel ‘structure-first’ view to help integrate both structural predictions and sequence annotations in manner that makes it easy for researchers make sense of the protein sequences they are working with.

The PSIPRED workbench remains a popular and well-used bioinformatics resource for researchers across the globe. In acknowledgement of the impact and importance of our web server, the site was accepted as an Elixir Web Resource as part of the Elixir UK node in 2019 (37). This enables us to take part in the coordination of services and life science research across the UK and Europe. This will help us to continue to develop and fund the service in the years to come.

## Data Availability

The web server is available at http://bioinf.cs.ucl.ac.uk/psipred/. Our principal code repository is available at https://github.com/psipred.
